# Expression and tissue distribution analysis of vimentin and transthyretin proteins associated with coat colors in sheep (*Ovis aries*)

**DOI:** 10.5713/ab.23.0111

**Published:** 2023-06-23

**Authors:** Zhihong Yin, Zhisheng Ma, Siting Wang, Shitong Hao, Xinyou Liu, Quanhai Pang, Xinzhuang Wang

**Affiliations:** 1 Postdoctoral Research Base, College of Veterinary Medicine, Henan Agricultural University, Zhengzhou 450046, China; 2 College of Animal Science and Veterinary Medicine, Henan Institute of Science and Technology, Xinxiang 453003, China; 3 College of Animal Science and Veterinary Medicine, Shanxi Agricultural University, Taigu 030801, China

**Keywords:** Coat Color, Sheep, Transthyretin, Vimentin

## Abstract

**Objective:**

Pigment production and distribution are controlled through multiple proteins, resulting in different coat color phenotypes of sheep.

**Methods:**

The expression distribution of vimentin (VIM) and transthyretin (TTR) in white and black sheep skins was detected by liquid chromatography–electrospray ionization tandem MS (LC–ESI–MS/MS), gene ontology (GO) statistics, immunohistochemistry, Western blot, and quantitative real time polymerase chain reaction (qRT-PCR) to evaluate their role in the coat color formation of sheep.

**Results:**

LC–ESI–MS/MS results showed VIM and TTR proteins in white and black skin tissues of sheep. Meanwhile, GO functional annotation analysis suggested that VIM and TTR proteins were mainly concentrated in cellular components and biological process, respectively. Further research confirmed that VIM and TTR proteins were expressed at significantly higher levels in black sheep skins than in white sheep skins by Western blot, respectively. Immunohistochemistry notably detected VIM and TTR in hair follicle, dermal papilla, and outer root sheath of white and black sheep skins. qRT-PCR results also revealed that the expression of VIM and TTR mRNAs was higher in black sheep skins than in white sheep skins.

**Conclusion:**

The expression of VIM and TTR were higher in black sheep skins than in white sheep skins and the transcription and translation were unanimous in this study. VIM and TTR proteins were expressed in hair follicles of white and black sheep skins. These results suggested that VIM and TTR were involved in the coat color formation of sheep.

## INTRODUCTION

The abundance of coat color in mammalian groups is an attractive biological phenomenon, which is ideal for studying and understanding the adaptive evolution of mammalian models. The color polymorphism of mammalian coat color plays an important role in avoiding natural enemies, predation, mating, and ultraviolet protection. The pigment process of mammalian coat color is determined by the quantity, quality, and distribution of melanin in the body [1]. The formation process of melanin is complex, including differentiation and maturation of melanocytes; morphogenesis of organelles, such as melanosomes; and anabolism and transport of melanin in melanocytes. However, each phase of cytochrome is accompanied by the participation of some important functional genes, which then forms a complex regulatory network of melanin metabolism by the interaction between genes. In turn, the different coat colors are helpful for mammals to adapt to different living environments. The research on the formation mechanism of different hair colors in mammals has always been important and a focus of genetics and evolutionary biology [2]. In mammals, long-term studies have found that mammalian hair color, eye color, and skin color are determined by the amount, quality, and distribution of melanin in the body. Moreover, mammals have two types of black color: eumelanin and melanin. Eumelanin is a dark insoluble polymer that could improve skin and hair’s black or brown appearance. Melanin is a yellow soluble polymer that makes the skin and hair appear red and yellow [3]. Melanin plays a key role in the formation of hair color in mammals. The formation of melanin has a complex regulatory mechanism, from the differentiation and maturation of melanocytes to the synthesis and transport of melanin involved in the regulation of multiple genes [4].

Vimentin (VIM) is a highly conserved type III intermediate filament protein, which belongs to the intermediate filament protein family. Changes in VIM lead to the rearrangement of the cytoskeleton and remodeling of the extracellular matrix, thus achieving the characteristics of mesenchymal cells and enhancing cell invasion [5]. VIM consists of three parts: N-terminal domain, central rod domain, and C-terminal domain. Phosphorylation and glycation site domains are mainly located at N- and C-terminals, which mainly regulate signal transduction. VIM is a substance with multiple effects, and it is involved in cell signal transduction, regulation of genomic DNA expression, formation of cell cohesion, regulation of cell migration, adhesion, and apoptosis [6,7]. VIM is also a dermal cell marker for hair follicles and is used to distinguish dermal cells from epidermal cells [7]. Research has demonstrated that VIM is expressed only in the outer root sheath of hair follicles and speculated to participate in the regulation of hair-follicle growth cycle by affecting the outer root sheath [7]. Furthermore, VIM could be used as one of the marker proteins for diagnosis of malignant melanoma, and it was found to be involved in the synthesis of melanin [8]. Transthyretin (TTR) is a tetramer with a molecular weight of 55 KD, mainly produced by the liver. Choroid plexus epithelium, visceral yolk sac endoderm, and retinal pigment epithelium could be synthesized by TTR [9]. Four TTR protein monomer molecules were combined with a T4 monomer molecule to form a polymer under physiological conditions. TTR protein polymers are unstable and easy to separate into monomers with TTR protein gene genetic mutation or other factors. The level of TTR protein was detected in serum, and it was found to be involved in operating the physiological function of animals [10]. In general, TTR appears in serum as a complex tetramer equilibrium, and even in small amounts as a monomer in normal body. The tetramer structure of TTR maintains its complex physiological work. Furthermore, TTR is prone to genetic mutations and could lead to the occurrence of various diseases.

The VIM and TTR were found in skins of white and black sheep through liquid chromatography–electrospray ionization tandem MS (LC–ESI–MS/MS). Consequently, VIM and TTR are probably involved in the coat color formation of sheep. In this study, white and black sheep skins were used to investigate the relationship of VIM and TTR with coat color and the contribution of the two to the color formation mechanism in sheep.

## MATERIALS AND METHODS

### Animals and sample collection

Rearing of sheep (*Ovis aries*) and collection of their skin samples were conducted in accordance with the International Guiding Principles for Biomedical Research Involving Animals (http://www.cioms.ch/frame1985textsofguidelines.html). Approval number of IACUC: LLSC2023031. Skin samples from six healthy 1-year-old white and black female sheep (three sheep per color) were selected from a sheep farm in Shanxi Agriculture University (Shanxi Province, China). The hair at the hindquarter of the sheep was carefully trimmed using fine dissecting scissors [11] to prevent bleeding. Three pieces of skin (8 mm in diameter for each piece) from the hindquarter of each sheep (three white and three black) were collected through punch skin biopsy under local anesthesia and immediately placed in liquid nitrogen for protein and RNA extraction. Additional white and black skin samples were fixed in Bouin’s solution for 24 h at 4°C and then extensively washed in 70% ethanol.

### LC–ESI–MS/MS

Total protein was extracted from skin samples (three white and three black) by using a tissue protein extraction kit (Boster, Wuhan, China) in accordance with the manufacturer’s instructions. The protein sample was digested with trypsin (Promega, Beijing, China), and the peptide fractionated by SCX chromatography through the AKTA purifier system (GE Healthcare, Chicago, IL, Country) was obtained. The dried peptide mixture was reconstituted and acidified with 2 mL buffer A (10 mM KH_2_PO_4_ in 25% ACN, pH 2.7) and loaded onto a 4.6×100 mm PolySULFOETHYL column (5 μm, 200 A; PolyLC Inc., Columbia, MD, USA). The peptides were eluted at a flow rate of 1 mL/min with a gradient of 0% to 10% buffer B (500 mM KCl, 10 mM KH_2_PO_4_ in 25% ACN, pH 2.7) for 2 min, 10% to 20% buffer B for 25 min, 20% to 45% buffer B for 5 min, and 50% to 100% buffer B for 5 min. The elution was monitored by absorbance at 214 nm, and fractions were collected every 1 min. The collected fractions (about 36 fractions) were finally combined into 15 pools and desalted on C18 Cartridges (Empore SPE Cartridges C18 [standard density], 7 mm bed I.D., volume 3 mL volume; Dionex, Sunnyvale, CA, USA). Each fraction was concentrated by vacuum centrifugation and reconstituted in 40 μL of 0.1% (v/v) trifluoroacetic acid. All samples were stored at −80°C until LC–MS/MS analysis. Moreover, each fraction sample was injected for nano LC–MS/MS analysis.

### Gene ontology functional item annotation

In the process of annotation, Blast2GO annotates qualified entries in gene ontology (GO) functional items extracted in mapping to target proteins by comprehensively considering the similarity between target sequence and alignment sequence, the reliability of GO item source, and the structure of GO directed acyclic graph. Here, the functional annotation of the target protein was preliminarily completed.

### Immunohistochemistry

Samples were dissected in sterile-filtered, ice-cold 1× phosphate-buffered saline (PBS); placed in fresh 4% paraformaldehyde; and fixed in 1× PBS supplemented with 0.3% Triton detergent (0.3% PBST). They were then washed in 0.3% PBST to remove fixative. Subsequently, the samples were blocked overnight at 4°C in 0.3% PBST supplemented with 1% Business Software Alliance (Fisher, BP1600-100), 1% normal donkey serum, and 1% normal goat serum (Jackson ImmunoResearch Laboratories 017-000-121 and 005-000-121). Afterwards, the sections were incubated in the presence of polyclonal rabbit anti-VIM (1:100 in PBS) or polyclonal rabbit anti-TTR (1:200 in PBS) at 4°C overnight. After the sections were washed, they were incubated in biotin-conjugated goat anti-rabbit IgG (Boster, China) at 37°C for 20 min. After being washed three times (3 min each time) in PBS, the sections were incubated with horseradish reddish peroxidase (Boster, China) at 37°C for 20 min. After being washed three times (3 min each time) with PBS, the sections were stained with 3,3″-diaminobenzidine (Boster, China) for 60 s. The sections were then counterstained with hematoxylin and washed with graded ethanol and dimethylbenzene. Afterwards, they were sealed with neutral balsam. Positive results were indicated by brown appearance. For negative controls, the primary antibody was replaced by non-immune bovine serum [12].

### Western blot analysis

Protein contents were measured using the BCA Protein Assay kit. Then, the protein samples were separated by sodium dodecyl sulfate-polyacrylamide gel electrophoresis (SDS-PAGE, 5% to 15% gels), and protein standards were used as the molecular weight marker. After electrophoresis was conducted, the proteins were transferred to PVDF membranes. The membranes were blocked with 5% nonfat-dried milk in PBS with 0.1% Tween 20 for 2 h and then incubated with primary antibodies overnight at 4°C. The primary antibodies were polyclonal rabbit anti-VIM antibody (Cat. No: bs-23063R; 1:1,000 in TBST; Beijing Biosynthesis Biotechnology Co., Beijing, China), polyclonal rabbit anti-TTR antibody (Cat. No: bs-23063R; 1:800 in TBST; Beijing Biosynthesis Biotechnology Co., China), and glyceraldehyde-3-phosphate dehydrogenase (GAPDH) (Cat. No: bs-2188R; 1:3,000 in TBST; Beijing Biosynthesis Biotechnology Co., China). After the primary antibodies were incubated overnight, the membranes were washed five times in PBS with 0.1% Tween 20 for 6 min, incubated with biotin-conjugated secondary antibodies for 1 h with gentle shaking, and washed again in PBS with 0.1% Tween 20. Blots were visualized by ECLTM (Bio-Rad, Hercules, CA, USA). Protein expression was quantified with ImageJ2x software.

### Quantitative real time polymerase chain reaction analysis

Total RNA was isolated from cells by using TRIzol reagent (Invitrogen Life Technologies, Carlsbad, CA, USA), following the manufacturer’s instructions. The total RNA concentration was measured with a microreader (BioTek Instruments Inc., Winooski, VT, USA) at 260/280 nm. Afterwards, 1 μg of total RNA was dissolved in 80 μL of 0.1% diethyl pyrocarbonate treated-deionized water for cDNA synthesis. The total RNA had a final volume of 20 μL. The SYBR Green PCR Master Mix kit (Life Technologies, Carlsbad, CA, USA) and Mx3000P qPCR System (Agilent Technologies, Santa Clara, CA, USA) were used to perform quantitative real time polymerase chain reaction (qRT-PCR). The reaction mixture comprised 1 μL of cDNA, 10 μL of 2× SYBR Green Master Mix, and the appropriate forward and reverse primers in a final volume of 20 μL. The PCR conditions were as follows: 95°C for 30 min, followed by 40 cycles of 95°C for 5 s, 60°C for 20 s, and 72°C for 20 s. The primers are listed in Table 1. Fluorescent signals were measured at the annealing/extension step, and each sample from each group was analyzed in duplicate for each tested gene. The qRT-PCR reaction was performed on a thermal cycler (C1000; BIO RAD, Hercules, CA, USA). The results of qRT-PCR were analyzed by 2^−ΔΔ^^CT^ method.

### Statistical analysis

LC–ESI–MS/MS data were analyzed by searching the MASCOT engine (Matrix Science, London, UK; version 2.2) embedded into Proteome Discoverer 1.4 (Thermo Electron, San Jose, CA, USA) against the *O. aries* sequence database (Uniprot *O. aries*). GO functional annotation and data analysis was conducted on Blast2GO application software. All data were analyzed using SPSS statistical package version 17.0 (SPSS Inc., Chicago, IL, USA). All results were expressed as mean ±standard deviation and analyzed using Student’s *t*-test and one-way analysis of variance.

## RESULTS

### LC–ESI–MS/MS analysis

**Figure 1:**
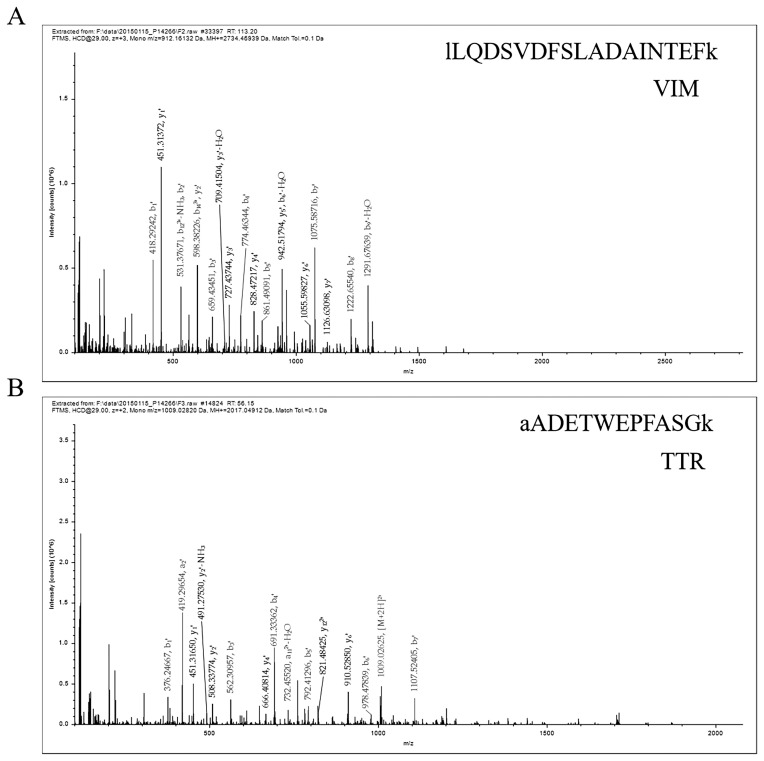
Representative MS/MS spectrum showing the peptide protein. (A) The peptide of vimentin (VIM) protein. (B) The peptide of transthyretin (TTR) protein.

The protein was analyzed by LC–ESI–MS/MS, and the information of VIM and TTR protein peptides was obtained, as shown in Figure 1A and B. The results showed that the peptide sequences were EMEENFSVEAANYQDTIGR and AADETWEPFASGK, and the database found that VIM and TTR proteins were expressed in white and black skin tissues of sheep.

### Gene ontology functional annotation analysis

**Figure 2:**
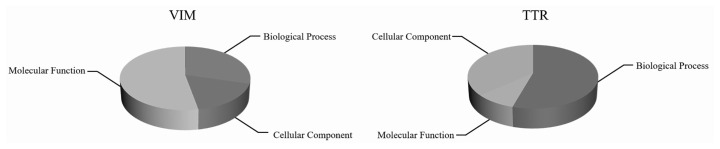
The classfication of gene ontology analysis.

Enrichment analysis of VIM and TTR proteins was conducted in white and black sheep skin tissues by GO classification. As shown in Figure 2, the function of VIM and TTR proteins were mainly concentrated in cellular components and biological process, respectively. The functional items of cell components mainly included plasma membrane, peroxisome, cell death, cytoskeleton, RNA binding, and cell differentiation. Meanwhile, the functional items of biological process mainly included small molecule metabolic process, extracellular space, signal transduction, lipid metabolic process, extracellular matrix organization, and cellular amino-acid metabolic process. Bioinformatics analysis showed that VIM and TTR proteins were involved in cell function in white and black sheep skin tissues.

### Immunohistochemistry of VIM and TTR proteins in white and black sheep

**Figure 3:**
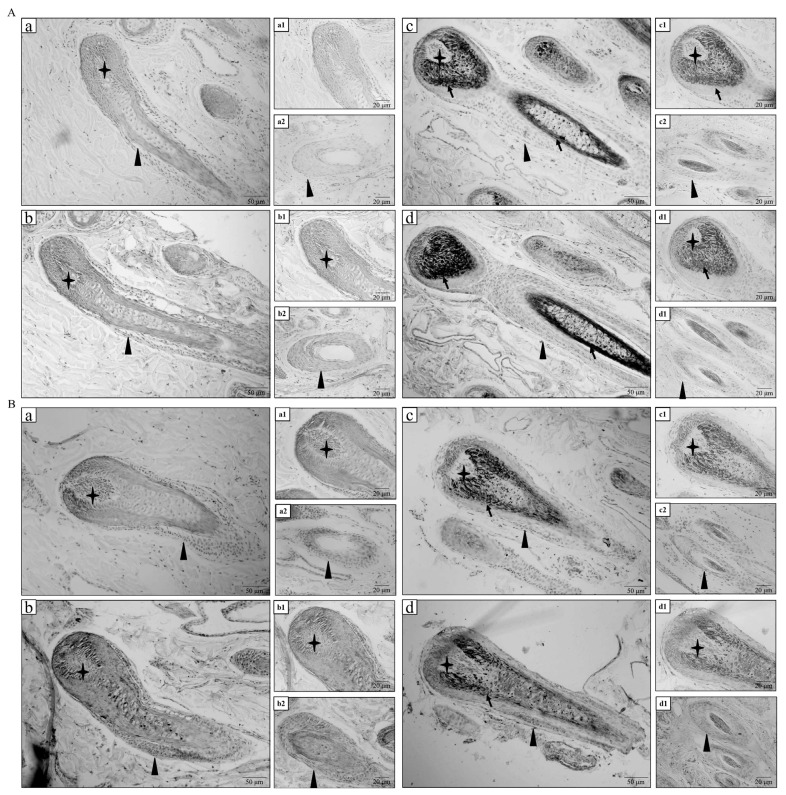
Immunohistochemistry analysis of protein expression levels of VIM and TTR in white and black sheep. (A) Immunohistochemistry analysis of VIM in white and black sheep. (a) Negative control of VIM in hair follicle of white sheep skin; (a1) Negative control of VIM in dermis papilla of white sheep skin; (a2) Negative control of VIM in outer root sheath of white sheep skin. (b) Positive VIM expression in hair follicle of white sheep skin; (b1) Positive VIM expression in dermis papilla of white sheep skin; (b2) Positive VIM expression in outer root sheath of white sheep skin. (c) Negative control of VIM in hair follicle of black sheep skin; (c1) Negative control of VIM in dermis papilla of black sheep skin; (c2) Negative control of VIM in outer root sheath of black sheep skin. (d) Positive VIM expression in hair follicle of black sheep skin; (d1) Positive VIM expression in dermis papilla of black sheep skin; (d2) Positive VIM expression in outer root sheath of black sheep skin. (B) Immunohistochemistry analysis of TTR in white and black sheep. (a) Negative control of TTR in hair follicle of white sheep skin; (a1) Negative control of TTR in dermis papilla of white sheep skin; (a2) Negative control of TTR in outer root sheath of white sheep skin. (b) Positive TTR expression in hair follicle of white sheep skin; (b1) Positive TTR expression in dermis papilla of white sheep skin; (b2) Positive TTR expression in outer root sheath of white sheep skin. (c) Negative control of TTR in hair follicle of black sheep skin; (c1) Negative control of TTR in dermis papilla of black sheep skin; (c2) Negative control of TTR in outer root sheath of black sheep skin. (d) Positive TTR expression in hair follicle of black sheep skin; (d1) Positive TTR expression in dermis papilla of black sheep skin; (d2) Positive TTR expression in outer root sheath of black sheep skin. The ✦ shows hair papilla; the ▲ shows outer root sheath; the black arrow shows melanin grain; scale (bar = 50 μm and bar = 20 μm). VIM, vimentin; TTR, transthyretin.

White and black sheep skins were analyzed through immunohistochemistry to investigate the distribution of VIM and TTR proteins. The immunohistochemistry results showed that VIM protein was mainly expressed in the hair papilla, outer root sheath, and inner root sheath of hair follicle in white sheep (Figures 3A-b1 and b2). Meanwhile, VIM protein was mainly expressed in the outer and inner root sheaths of hair follicle in black sheep (Figures 3A-d1 and d2). As shown in Figures 3B-b1 and b2, TTR protein was expressed in the hair papilla, outer root sheath, inner root sheath, and hair shaft of hair follicle in white sheep. As shown in Figures 3B-d1 and d2, TTR protein was also expressed in the hair papilla, outer root sheath, and hair shaft of hair follicle in black sheep. No positive expression was observed in the negative control group (Figures 3A-a1 and a2, 3A-c1 and c2, 3B-a1 and a2, and 3B-c1 and c2). The immunohistochemistry results confirmed that VIM and TTR proteins were expressed in hair follicles of white and black sheep.

### Western blot analysis of VIM and TTR proteins

**Figure 4:**
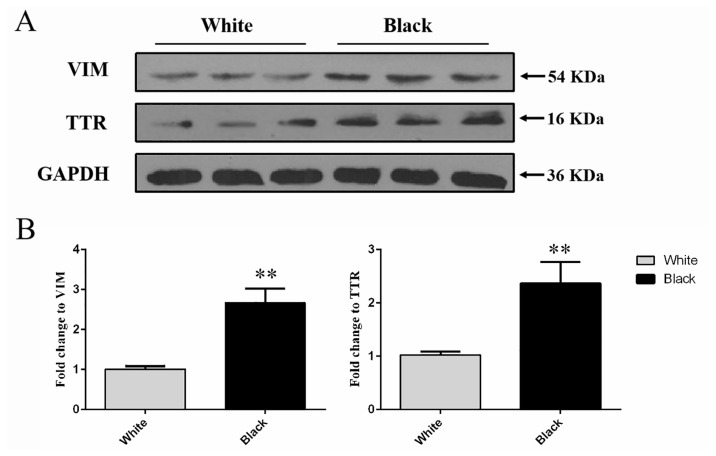
Western blot analysis of protein expression levels of VIM and TTR in white and black sheep skin. (A) Western blot results of VIM and TTR in white and black sheep skin. (B) Relative protein expression levels of VIM and TTR in white and black sheep skin. VIM, vimentin; TTR, transthyretin. Bars in panel represent mean±standard deviation (n = 3), ** p<0.01.

Western blot was conducted to analyze the protein level of VIM and TTR were detected at 53 and 16 kDa in the total protein extracted from the white and black skins (Figure 4A). The results showed that the protein expression level of VIM and TTR in black sheep skin was significantly higher that detected in white sheep skin respectively (p<0.01) (Figure 4B), which was preliminarily confirmed that VIM and TTR proteins were differentiated in different color skin.

### qRT-PCR analysis of VIM and TTR mRNA

**Figure 5:**
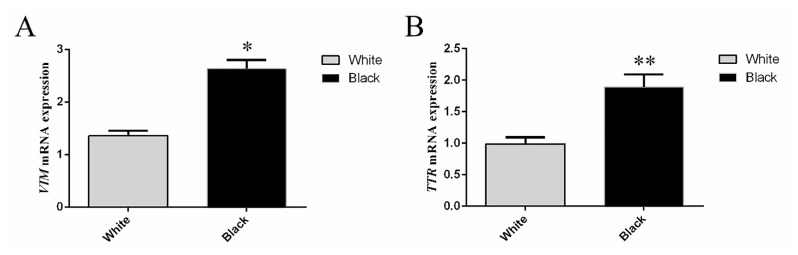
Relative expression of *VIM* and *TTR* mRNA in skin samples collected from the white and black sheep. (A) Relative expression of *VIM* mRNA in skin samples collected from the white and black sheep. (B) Relative expression of *TTR* mRNA in skin samples collected from the white and black sheep. Relative VIM and TTR expression were normalized relative to abundance of 18s. VIM, vimentin; TTR, transthyretin. Bars in each panel represent the mean±standard deviation (n = 3), * p<0.05, ** p<0.01.

The expression levels of VIM and TTR in sheep skins with different coat colors are shown in Figure 5. VIM and TTR were highly expressed in skin samples collected from sheep with black coat color, and the VIM relative expression of mRNA in black skin was 1.95 times than that in white skin, with significant difference (p<0.05). Moreover, the TTR relative expression of mRNA in black skin was 1.90 times than that in white skin, also with significant difference (p<0.01). The VIM and TTR mRNA expression levels were consistent with the protein level from each coat color.

## DISCUSSION

Coat color is one of the most important characteristics used to distinguish livestock breeds. Under the combined influence of gene regulation and environment, livestock exhibits the final coat color. Wool color is an important production trait of sheep [13,14]. The coat color of sheep is mainly white, black, brown, and patchy variegated [15]. White wool contains little or no fusomelanin, brown and black wool contains melanin or a combination of two pigments, and completely black sheep are rare. Therefore, in this study, pure white and black sheep were selected in the early stage, and different sheep hair color-related differential proteins were screened by LC–ESI–MS/MS technology. VIM and TTR potential coat color-related proteins were also screened out for verification and analysis in sheep.

Hair follicles are important accessory organs of the skin, and they are renewable. Hair follicles are composed of epithelial and dermal tissues and are processing plants for hair [16,17]. The size and shape of hair are determined by hair papillae, which consist of specialized fibroblasts at the base of the hair follicle. Meanwhile, hair color is determined by the pigment produced by melanocytes, which are scattered among stromal cells [18]. The stem cell groups in mammalian hair follicle have several types. Hair follicle depends on the timely and organized coordination among different stem cells to play a regenerative function [19,20]. The epithelial and melanin stem cells related to hair color distribute the projection and secondary hair stromal cells of hair follicle. Differentiated melanocyte stem cells transfer their self-produced melanin to neighboring epithelial cells during early hair follicle growth, and differentiated pigment cells undergo apoptosis as a small fraction of hair follicle degenerates during late hair follicle growth [21].

Hair color depends on the pigment production in hair follicle. Furthermore, hair follicles are divided into inner root sheath, outer root sheath, and fibrous sheath from the inside out. In this study, VIM and TTR were detected in the hair follicle, dermal papilla, and outer root sheath of white and black sheep skins. The localization revealed that VIM protein was perhaps associated with pigment formation of sheep skin. It is well-know that *KIT*, tyrosinase (*TYR*), tyrosinase protein 1 (*TYRP1*), agouti signaling protein (*ASIP*), and melanocortin receptor - 1 (*MCIR*) genes play pivotal role in regulating coat color. The studies revealed that RAD6B loss and the expression levels of VIM, MITF-M, melan A, and TYRP1 (a tyrosinase family member critical for melanin biosynthesis) were reduced, which regulating melanocyte development and pigmentation signaling in M14 cells [22]. More importantly, VIM protein was expressed only in the outer root sheath of hair follicles in Mongolian Cashmere goats [7]. Melanoblasts are located in the root sheath of hair follicle, and they migrate to the dermal papilla, which affects coat color formation. Therefore, VIM increased and simulated melanocytes to produce more pigments in the hair follicle of sheep, resulting in black coat color formation. TTR plays an important role in the plasma transport of thyroxine and retinol. In this study, TTR was detected in the hair follicle, hair papilla, and outer root sheath of white and black sheep skins. Thus, TTR possibly enters the hair follicle via the blood vessel of hair papilla and is may be involved in sheep pigmentation.

Further research confirmed that the expression of VIM and TTR proteins were higher in black tissue than in white tissue of sheep. Furthermore, the results were consistent at the protein and transcription levels. VIM expression has been confirmed to be higher in the yellow region and involved in intracellular pigment mobilization of East African cichlid fish [23]. The present study revealed that VIM have potential function of regulating coat color formation. Studies have proven that TTR protein expression was decreased in Marek’s disease-infected chickens, with iris discoloration and dark coat color [24]. However, which pigment synthesis factor and TTR affect to regulate the formation of hair color, it is not clear at present. From this study, we conjecture that TTR enter the hair follicle through vessels that distributed the dermal papilla that because of TTR exists in the blood, and may affects the pigment synthesis and the formation of hair color. In addition, VIM, TTR, and TYR genes were used to evaluate the diagnosis of melanoma, which suggested VIM and TYR involved in pigment synthesis, and the mechanism of action needs further study [25–27].

As a conclusion, VIM and TTR exhibited significantly higher protein and mRNA expression levels in black sheep skin than in white sheep skin. They were distributed in the hair papilla and outer root sheath of hair follicle in sheep. The results obviously suggested that the expression of VIM and TTR differed between the skin tissues of white and black sheep. We preliminarily proved that VIM and TTR proteins were involved in pigment synthesis to regulate the color coat of sheep.

## Figures and Tables

**Table 1:** Primer sequences and their corresponding polymerase chain reaction product sizes

Genes	Primer sequence (5′→3′)	Product size (bp)	Tm (°C)
*VIM*	F: GGATTTCTCTGCCTCTTCCA R: TCTCTGGTTTCCACCGTCTT	124	57.6
*TTR*	F: TTCCGTCTGCTCCTCCTTT R: AACACCTTCACACCCACATTC	149	58.2
*18s RNA*	F: GAAGGGCACCACCAGGAGT R: CAGACAAATCACTCCACCAA	131	59.9

VIM, vimentin; TTR, transthyretin.

F: Sense primers; R: Antisense primer.

## SUPPLEMENTARY MATERIAL

The dataset generated during the current study is available from the corresponding author (Xinzhuang Wang) on reasonable request.

## References

[1] Ohbayashi N, Fukuda M (2020). Recent advances in understanding the molecular basis of melanogenesis in melanocytes. F1000Res.

[2] Pillaiyar T, Namasivayam V, Manickam M, Jung SH (2018). Inhibitors of melanogenesis: An updated review. J Med Chem.

[3] Lamoreux ML, Wakamatsu K, Ito S (2001). Interaction of major coat color gene functions in mice as studied by chemical analysis of eumelanin and pheomelanin. Pigment Cell Res.

[4] Vandamme N, Berx G (2019). From neural crest cells to melanocytes: Cellular plasticity during development and beyond. Cell Mol Life Sci.

[5] Huang R, Zong X (2017). Aberrant cancer metabolism in epithelial-mesenchymal transition and cancer metastasis: Mechanisms in cancer progression. Crit Rev Oncol Hematol.

[6] Sehati N, Sadeghie N, Mansoori B, Mohammadi A, Shanehbandi D, Baradaran B (2020). MicroRNA-330 inhibits growth and migration of melanoma A375 cells: In vitro study. J Cell Biochem.

[7] Rile N, Liu Z, Gao L (2018). Expression of vimentin in hair follicle growth cycle of inner mongolian cashmere goats. BMC Genomics.

[8] Wurth L, Papasaikas P, Olmeda D (2016). UNR/CSDE1 drives a post-transcriptional program to promote melanoma invasion and metastasis. Cancer Cell.

[9] Minnella AM, Rissotto R, Antoniazzi E (2021). Ocular involvement in hereditary amyloidosis. Genes (Basel).

[10] Finn JD, Smith AR, Patel MC (2018). A single administration of CRISPR/Cas9 lipid nanoparticles achieves robust and persistent in vivo genome editing. Cell Rep.

[11] Tian X, Jiang J, Fan R (2012). Identification and characterization of microRNAs in white and brown alpaca skin. BMC Genomics.

[12] Yin Z, Ge Y, Ning H (2019). Expression and tissue distribution analysis of angiotensin II in sheep (Ovis aries) skins associated with white and black coat colors. Acta Histochem.

[13] Gebreselassie G, Berihulay H, Jiang L, Ma Y (2020). Review on genomic regions and candidate genes associated with economically important production and reproduction traits in sheep (Ovies aries). Animals (Basel).

[14] Mortimer SI, Hatcher S, Fogarty NM (2017). Genetic correlations between wool traits and carcass traits in merino sheep. J Anim Sci.

[15] McManus C, Louvandini H, Gugel R (2011). Skin and coat traits in sheep in brazil and their relation with heat tolerance. Trop Anim Health Prod.

[16] Batcher K, Varney S, Affolter VK, Friedenberg SG, Bannasch D (2022). An SNN retrocopy insertion upstream of GPR22 is associated with dark red coat color in poodles. G3 (Bethesda).

[17] Grymowicz M, Rudnicka E, Podfigurna A (2020). Hormonal effects on hair follicles. Int J Mol Sci.

[18] Park AM, Khan S, Rawnsley J (2018). Hair Biology: Growth and pigmentation. Facial Plast Surg Clin.

[19] Robinson KC, Fisher DE (2009). Specification and loss of melanocyte stem cells. Semin Cell Dev Biol.

[20] Gentile P, Garcovich S (2019). Advances in regenerative stem cell therapy in androgenic alopecia and hair loss: Wnt pathway, growth-Factor, and mesenchymal stem cell signaling impact analysis on cell growth and hair follicle development. Cells.

[21] Chang CY, Pasolli HA, Giannopoulou EG (2013). NFIB is a governor of epithelial-melanocyte stem cell behaviour in a shared niche. Nature.

[22] Sarma A, Gajan A, Kim S (2021). RAD6B loss disrupts expression of melanoma phenotype in part by inhibiting WNT/β-catenin signaling. Am J Pathol.

[23] Ahi EP, Lecaudey LA, Ziegelbecker A (2020). Comparative transcriptomics reveals candidate carotenoid color genes in an east african cichlid fish. BMC Genomics.

[24] Chen X, Hu X, Yu C, Qian K, Ye J, Qin A (2014). Differential protein analysis of chicken skin infected with Marek’s disease virus. Acta Virol.

[25] Weeraratna AT, Gorospe M (2016). UNRelenting translation UNRestrains melanoma migration. Cancer Cell.

[26] Pagliarello C, Magi S, Mazzoni L, Stanganelli I (2021). Proportion of thick versus thin melanomas as a benchmarking tool. J Clin Med.

[27] Smedley RC, Sebastian K, Kiupel M (2022). Diagnosis and prognosis of canine melanocytic neoplasms. Vet Sci.

